# Supplementary figures and images for: TTSS-2 virulence drives inflammatory destruction of the gut epithelial barrier and modulates inflammatory response profiles in the Salmonella-infected mouse gut
Source: PLoS Pathog. 2025 Dec 10;21(12):e1013733. doi: 10.1371/journal.ppat.1013733 (PMC12694825; doi:10.1371/journal.ppat.1013733)

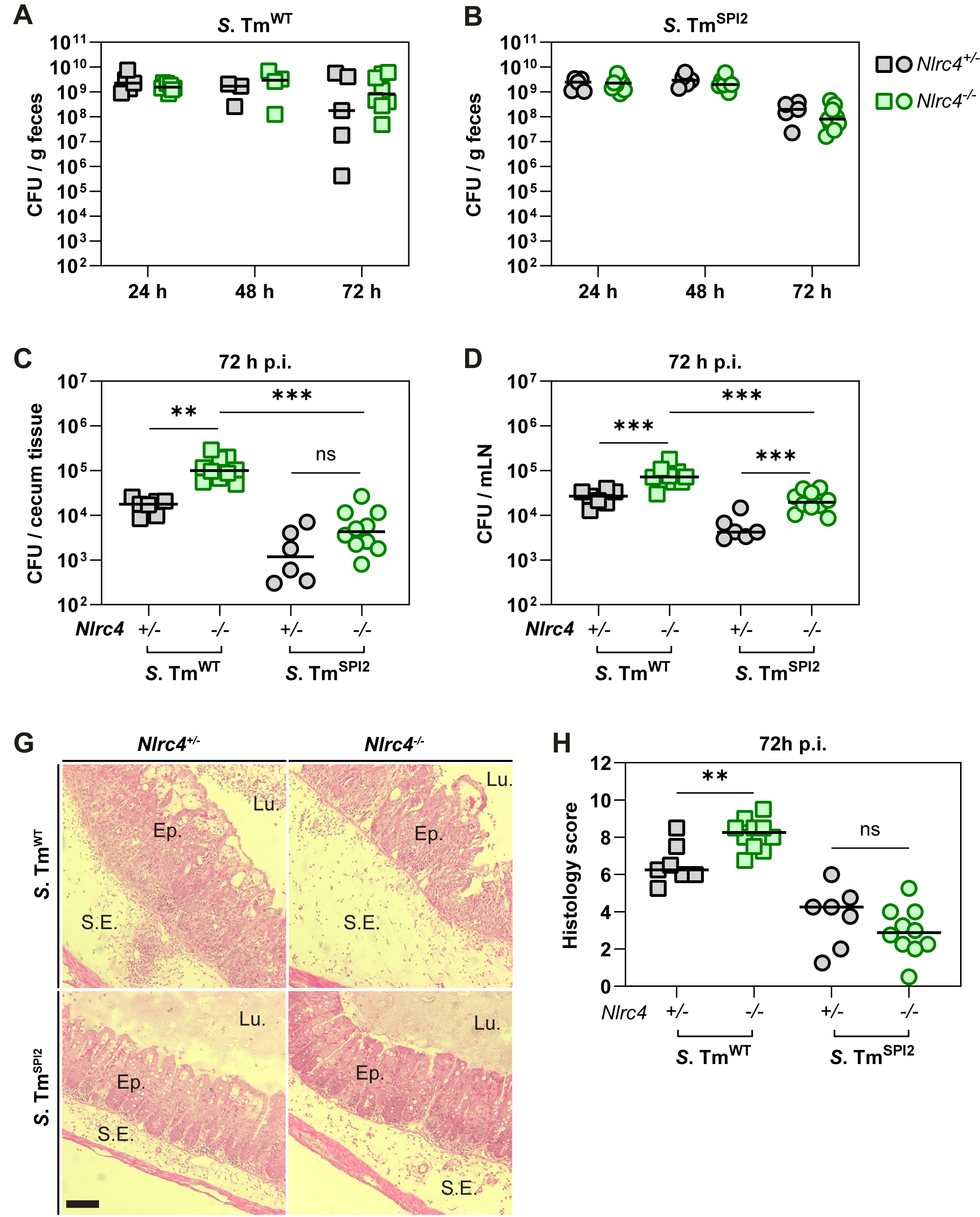

Supplement: S1 Fig — (a–f) Experimental setup as described in Fig 1. (a, b) Fecal S. TmWT (a) and S. TmSPI2 (b) pathogen loads as determined using MacConkey plates with selective antibiotics. (c) S. TmWT and S. TmSPI2 pathogen loads in cecum tissue and (d) in mesenteric lymph nodes. (g) Representative images of H&E-stained cecum tissue sections Lu. lumen, S.E. submucosa edema. Scale bars: 100 μm. (h) Quantification of pathology scores based on histological evaluation of (g). (a-d, h) Each point represents average of one mouse. Line at median. Mann-Whitney U test (*p < 0.05, **p < 0.01, ns – not significant). (TIF) [file ppat.1013733.s001.tif]

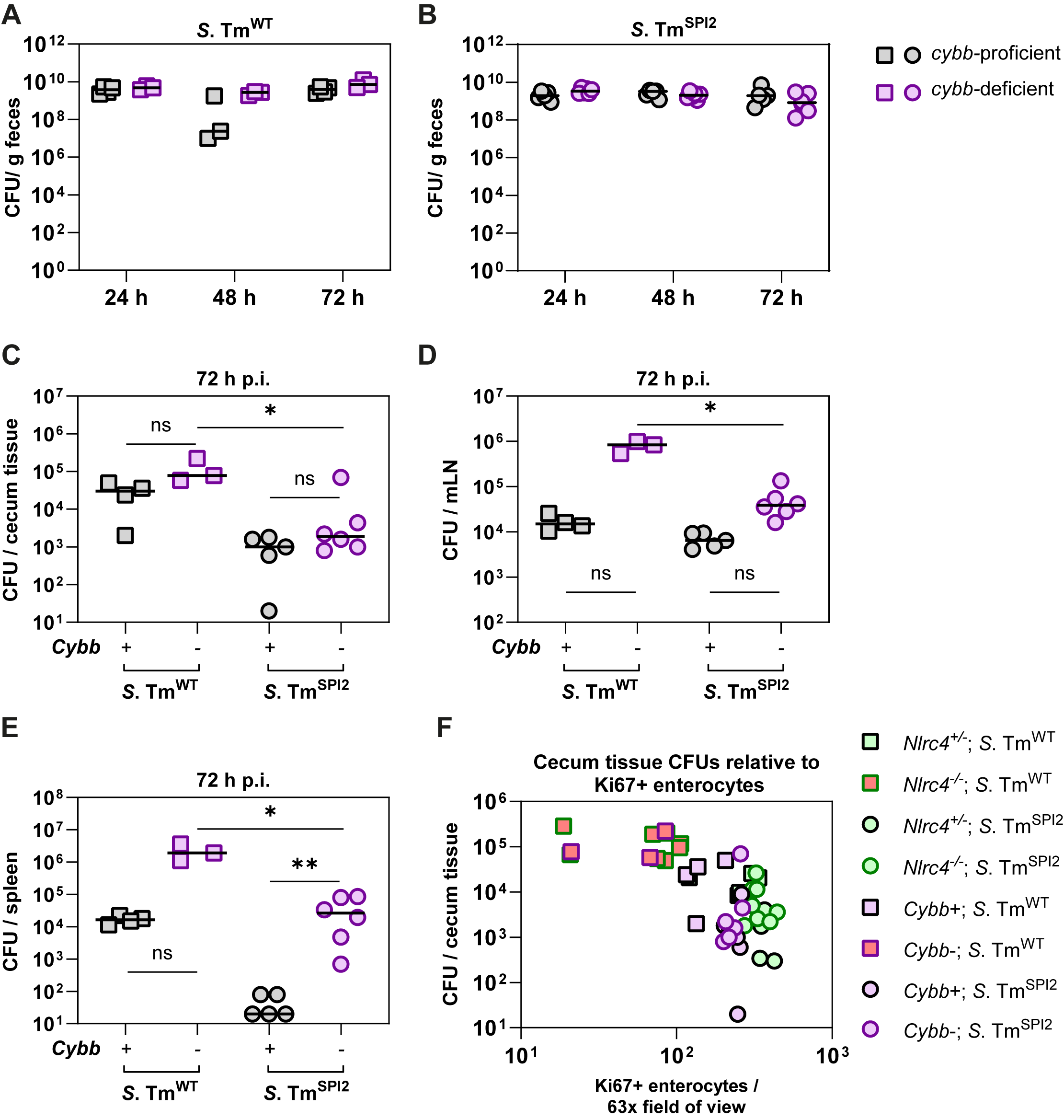

Supplement: S2 Fig — (a–f) Experimental setup as described in Fig 2. (a, b) Fecal S. TmWT (a) and S. TmSPI2 (b) pathogen loads as determined using MacConkey plates with selective antibiotics. (c) S. TmWT and S. TmSPI2 pathogen loads in cecum tissue, (d) in mesenteric lymph nodes and (e) in the spleen. (f) Cecum tissue S. Tm loads plotted over Ki67 + enterocytes/63X field of view. Plot contains all mice from Figs 1 and 2. Dots filled with red colour belong to mice with reduced regeneration capacity in Figs 1C and 2C. (a-f) Each point represents average of one mouse. Line at median. Mann-Whitney U test (*p < 0.05, **p < 0.01, ns – not significant). (TIF) [file ppat.1013733.s002.tif]

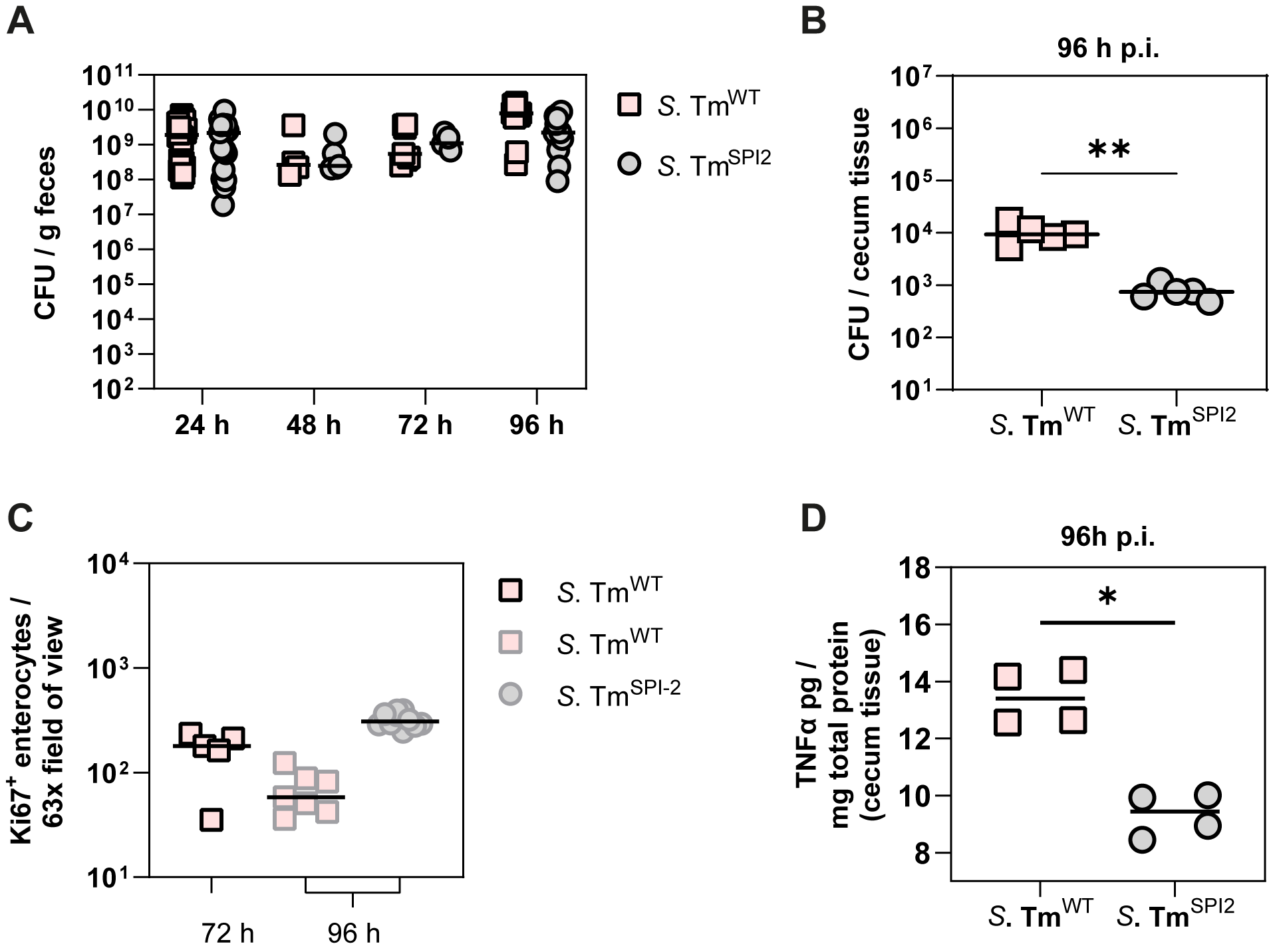

Supplement: S3 Fig — (a–d) Experimental setup as described in Fig 3. (a) Fecal S. TmWT and S. TmSPI2 pathogen loads as determined using MacConkey plates with selective antibiotics. (b) S. TmWT and S. TmSPI2 pathogen loads in cecum tissue. (c) Dots with grey border are re-plotted from Fig 3E for reference. Microscopy-based quantification of Ki-67 positive epithelial cells per 63x field of view (72 h p.i. and 96 h p.i.) (d) TNFα concentrations were quantified by ELISA and expressed relative to total protein content (pg/mg), as determined by Bradford assay. (a-d) Each point represents average of one mouse. Line at median. Mann-Whitney U test (*p < 0.05, **p < 0.01, ns – not significant). (TIF) [file ppat.1013733.s003.tif]

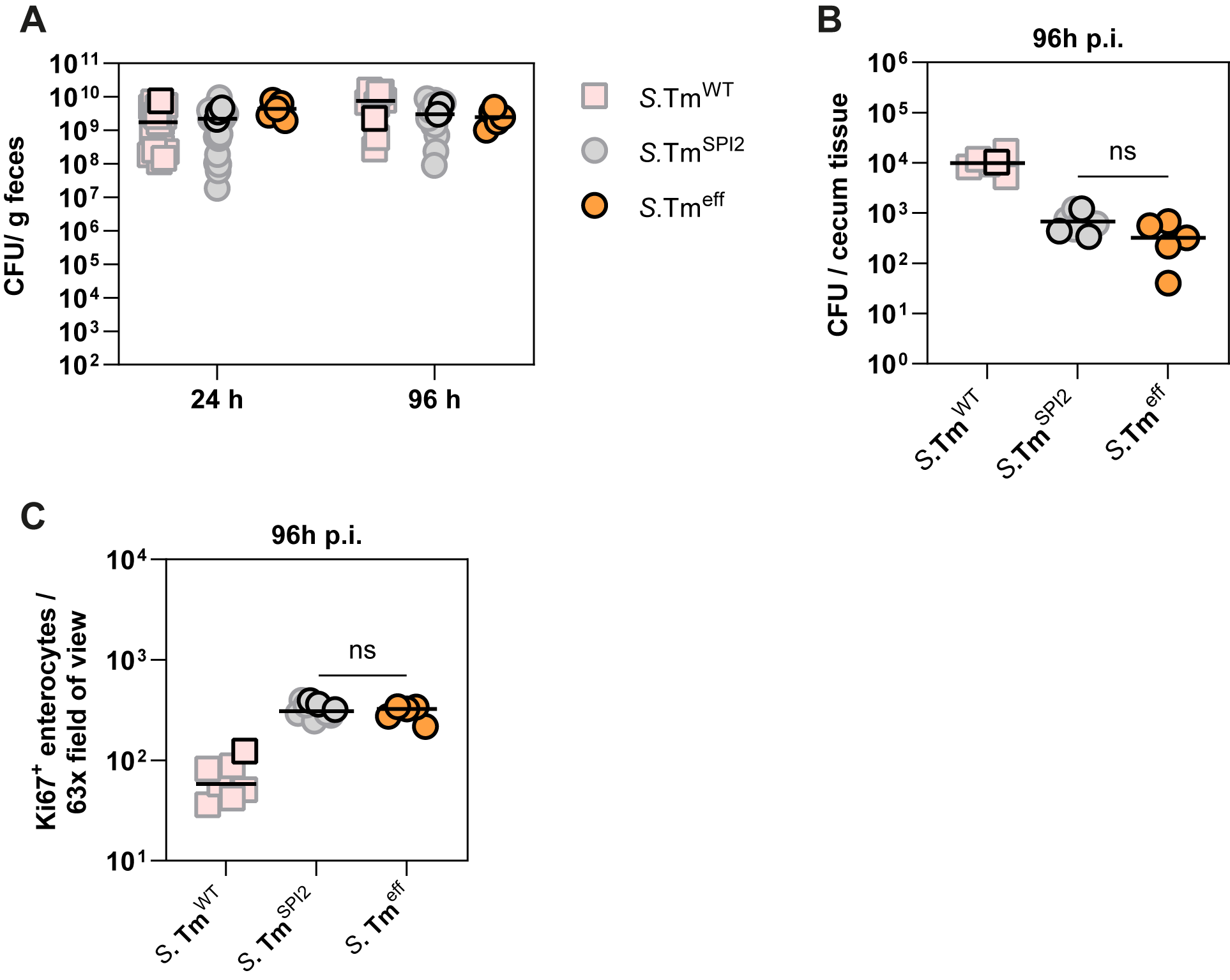

Supplement: S4 Fig — (a–c) Experimental setup as described in Fig 3. Dots with grey border are re-plotted from S3 Fig for reference. (a) Fecal S. TmWT, S. TmSPI2 and S. Tmeff pathogen loads as determined using MacConkey plates with selective antibiotics. (b) S. TmWT, S. TmSPI2 and S. Tmeff pathogen loads in cecum tissue. (c) Microscopy-based quantification of Ki-67 positive epithelial cells per 63x field of view (a-c) Each point represents average of one mouse. Line at median. Mann-Whitney U test (*p < 0.05, **p < 0.01, ns – not significant). (TIF) [file ppat.1013733.s004.tif]

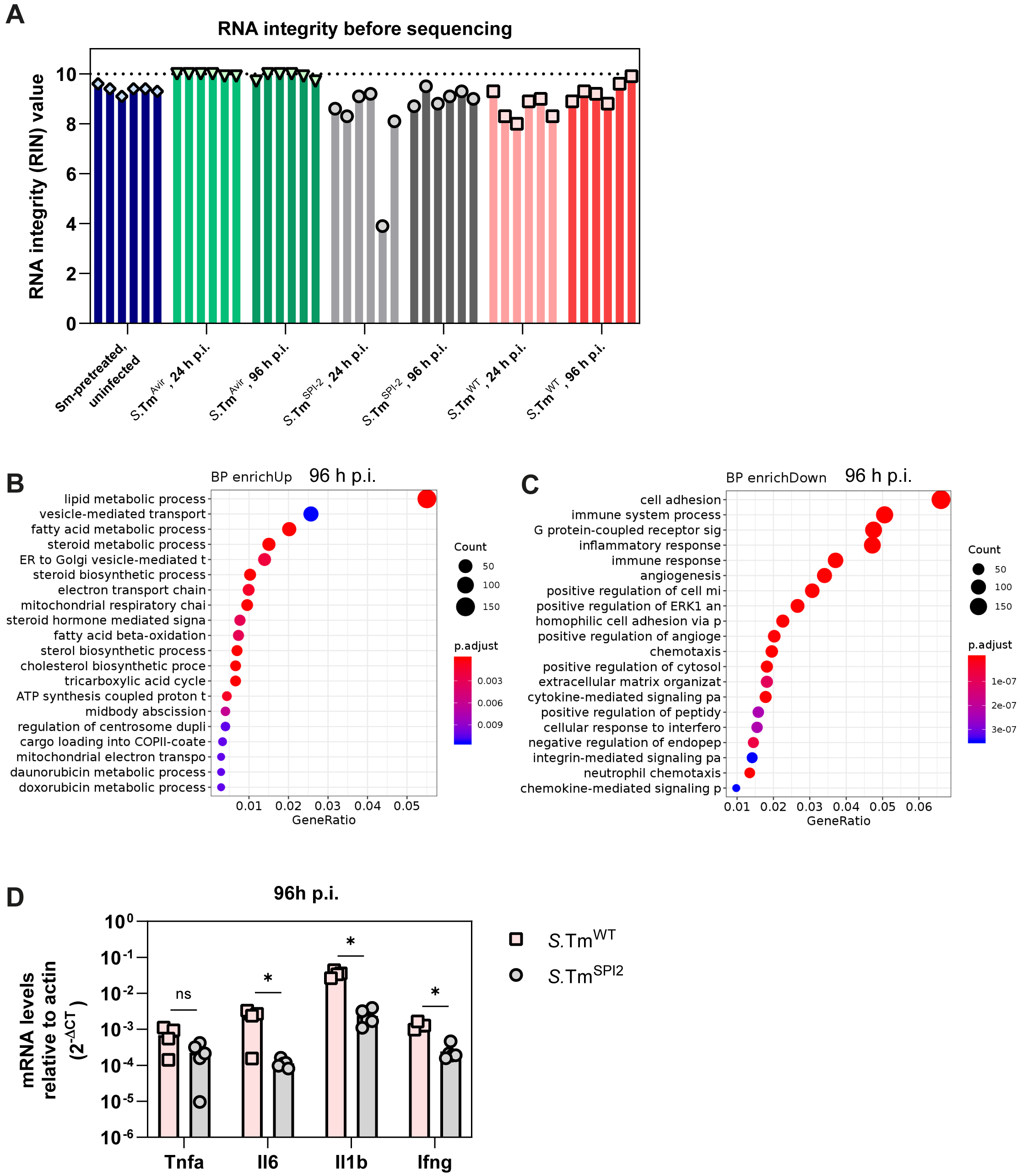

Supplement: S5 Fig — (a) RIN values of individual RNA samples before library preparation. All samples exceeded the quality threshold (RIN ≥ 8) required for sequencing, except for one sample with RIN = 3.9. (b, c) Dot plot of enriched Gene Ontology (GO) biological processes among differentially expressed genes (DEGs) between S. TmSPI2 and S. TmWT. The x-axis represents the gene ratio (number of DEGs associated with a GO term divided by the total number of input DEGs). Dot size corresponds to the number of genes in each term, and colour indicates statistical significance (adjusted p-value). (b) 96 h p.i. Only upregulated genes. (c) 96 h p.i. Only downregulated genes. (d) 96 h p.i. Quantification of mRNA expression levels in the cecal mucosa by RT-qPCR. Results are presented relative to b-actin mRNA levels. (TIF) [file ppat.1013733.s005.tif]

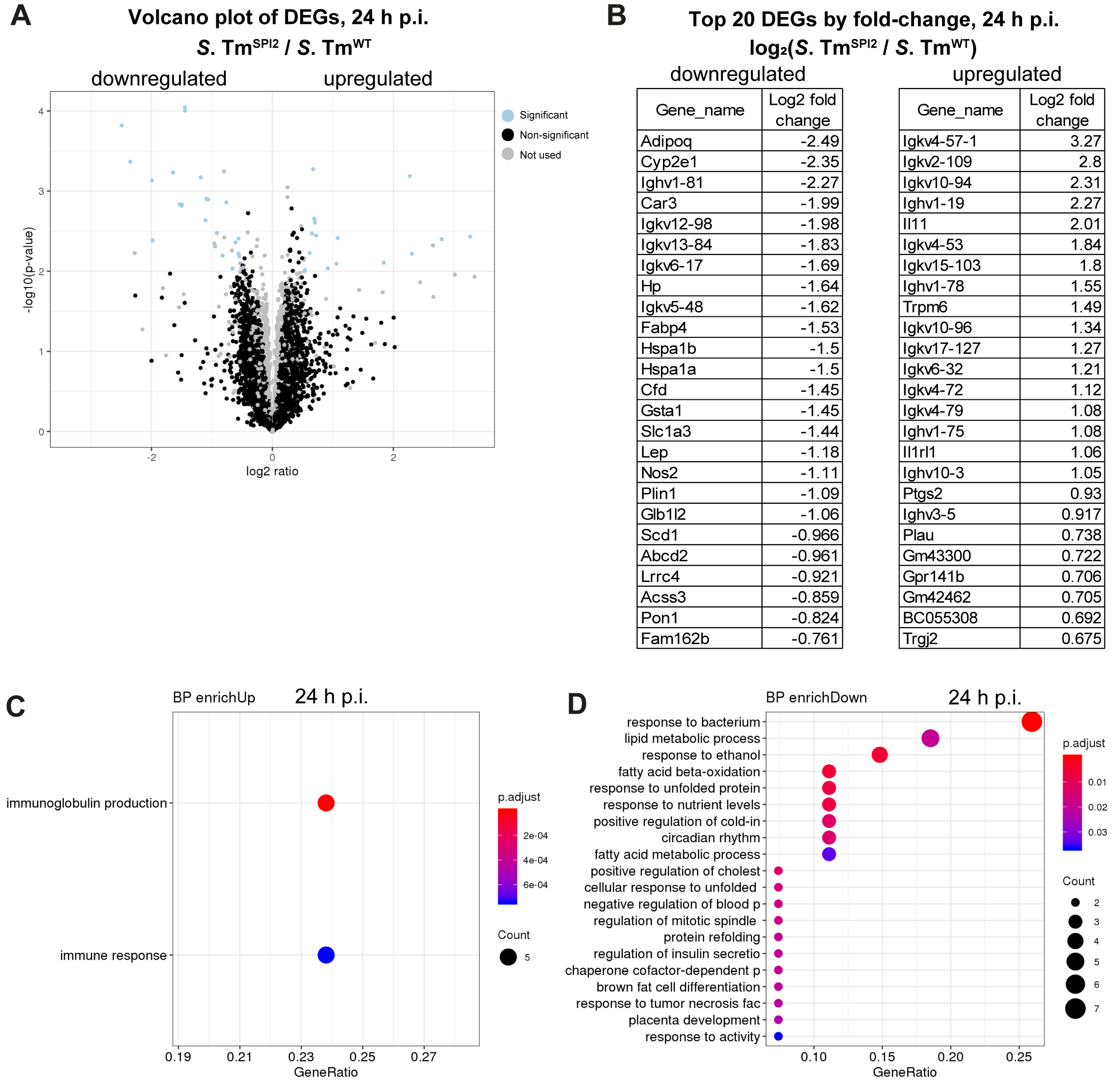

Supplement: S6 Fig — (a) Volcano plot showing the DEGs between S. TmSPI2 and S. TmWT at 24 h p.i. The x-axis represents the log₂ fold change in gene expression (S. TmSPI2 vs. S. TmWT), and the y-axis shows the –log₁₀ of the p-value. (b) List of the top 20 most strongly upregulated and top 20 most strongly downregulated genes between S. TmSPI2 and S. TmWT at 24 h p.i., selected from the 500 most significant DEGs. Genes are ranked by absolute log₂ fold change. Positive fold change values indicate higher relative expression in S. TmSPI2, and negative values indicate higher relative expression in S. TmWT. (c, d) Dot plot of enriched Gene Ontology (GO) biological processes among differentially expressed genes (DEGs) between S. TmSPI2 and S. TmWT. The x-axis represents the gene ratio (number of DEGs associated with a GO term divided by the total number of input DEGs). Dot size corresponds to the number of genes in each term, and colour indicates statistical significance (adjusted p-value). (c) 24 h p.i. Only upregulated genes. (d) 24 h p.i. Only downregulated genes. (TIF) [file ppat.1013733.s006.tif]

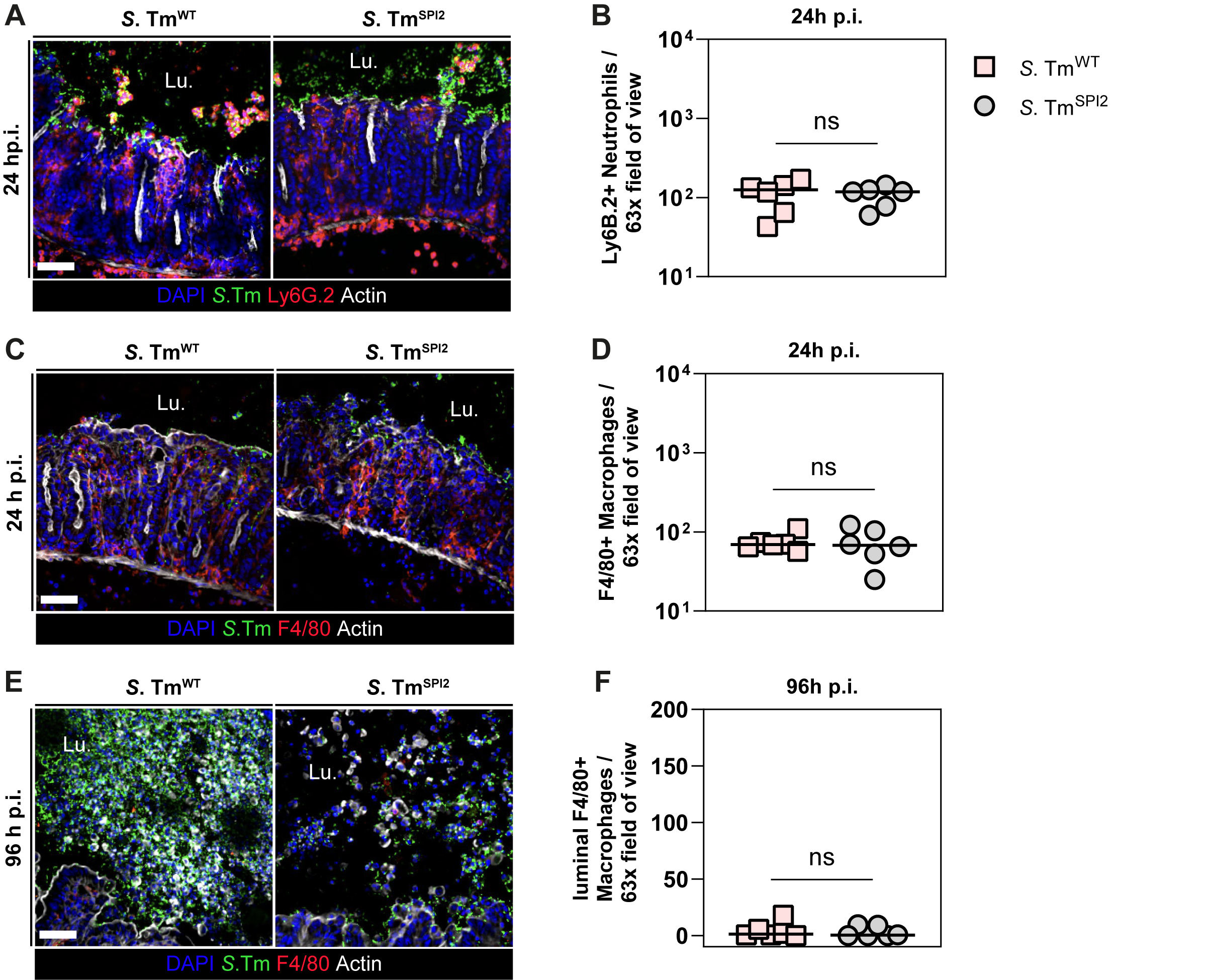

Supplement: S7 Fig — (a) Representative micrographs of cecum tissue sections stained for neutrophil marker Ly6B.2 at 24h p.i. Lu.: Lumen. Scale bar: 50 µm. (b) Microscopy-based quantification of Ly6B.2 positive cells per 63x field of view in (a). (c) Representative micrographs of cecum tissue sections, stained for macrophage marker F4/80 at 24h p.i. Lu.: Lumen. Scale bar: 50 µm. (d) Microscopy-based quantification of F4/80 positive cells per 63x field of view in (c). (e) Representative micrographs of cecum lumen sections, stained for macrophage marker F4/80 at 96h p.i. Lu.: Lumen. Scale bar: 50 µm. (f) Microscopy-based quantification of F4/80 positive cells per 63x field of view in (e). (b, d, f) Each point represents average of one mouse. Line at median. Mann-Whitney U test (*p < 0.05, **p < 0.01, ns – not significant). (TIF) [file ppat.1013733.s007.tif]

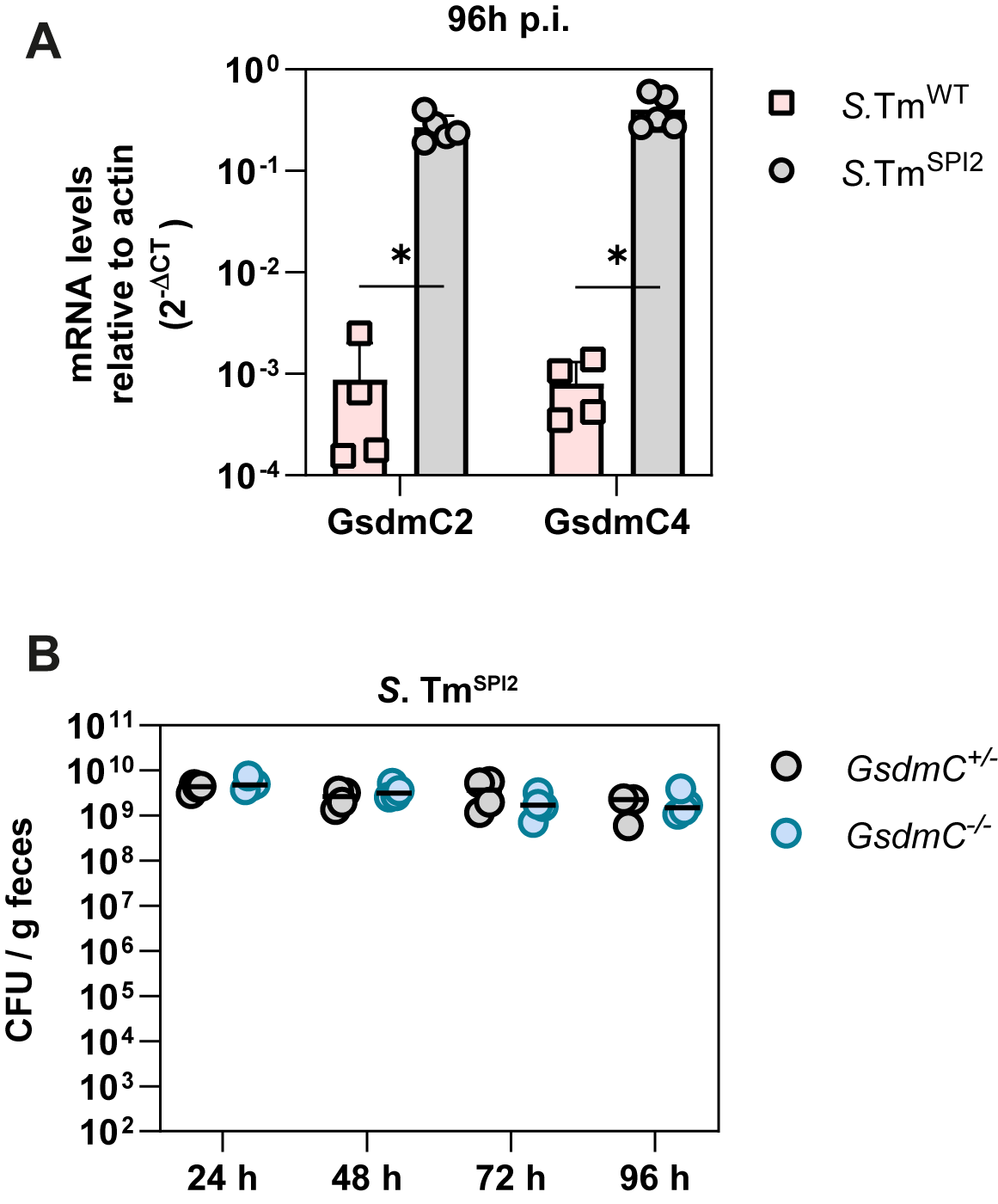

Supplement: S8 Fig — (a) Quantification of mRNA expression levels in the cecal mucosa by RT-qPCR of S. TmSPI2- and S. TmWT-infected C57BL/6 J mice at 96 h p.i. Results are presented relative to b-actin mRNA levels. (b) Experimental setup as described in Fig 5. Fecal S. TmSPI2 pathogen loads as determined using MacConkey plates with selective antibiotics. (TIF) [file ppat.1013733.s008.tif]

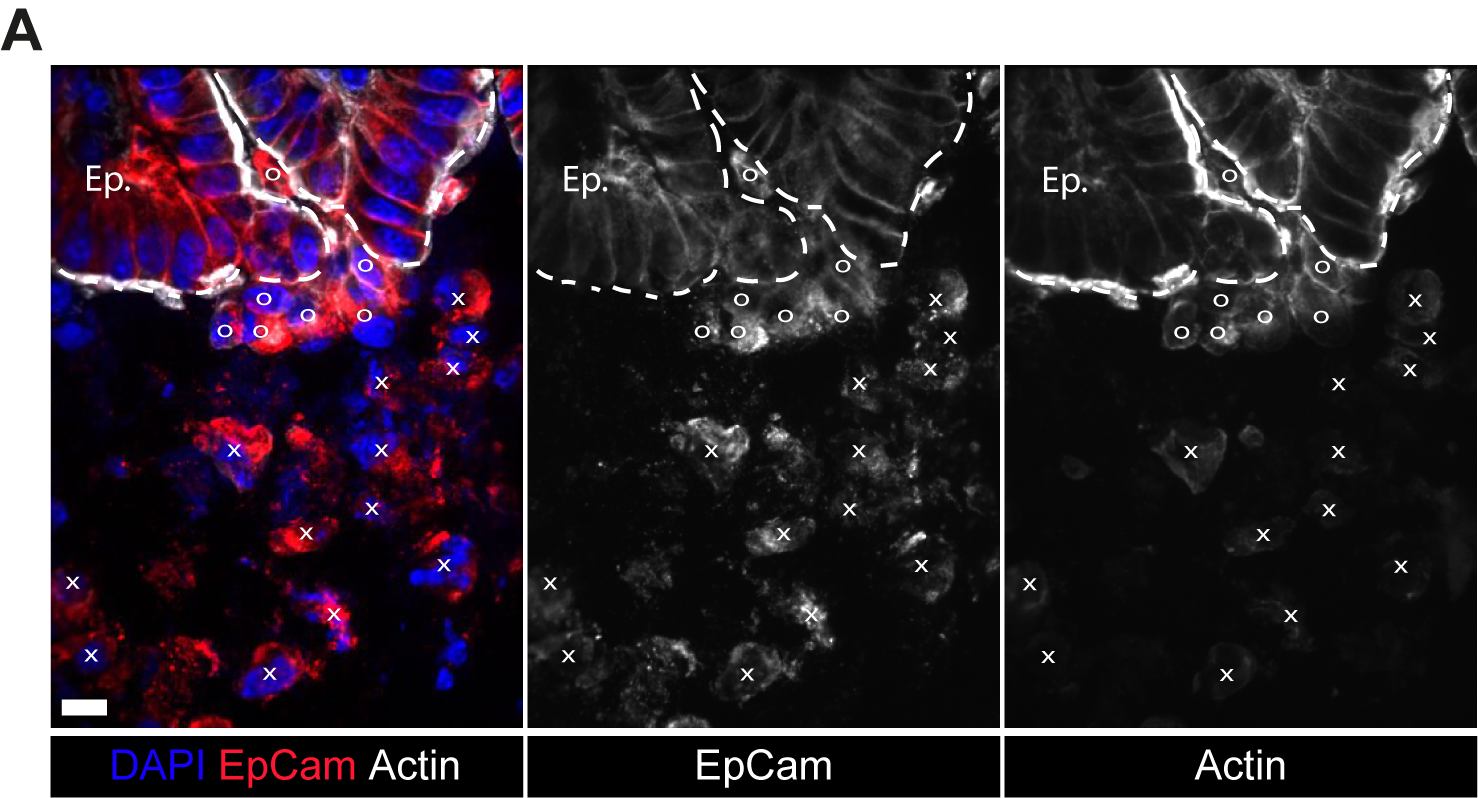

Supplement: S9 Fig — (a) Representative micrographs of cecum lumen sections, stained for EpCam, Actin and DAPI. Ep.: Epithelium. Dashed line: Actin brush border that indicates the outer line of the epithelium. O: Intestinal epithelial cells that are classified as non-dislodged for quantification as they are in contact with the epithelial layer. X: Intestinal epithelial cells that are classified as dislodged as they are located in the lumen without attachment to the epithelial layer. Scale bar: 20 µm. (TIF) [file ppat.1013733.s009.tif]
