# Supplementary material for: TTSS-2 virulence drives inflammatory destruction of the gut epithelial barrier and modulates inflammatory response profiles in the Salmonella-infected mouse gut
Source: PLoS Pathog. 2025 Dec 10;21(12):e1013733. doi: 10.1371/journal.ppat.1013733 (PMC12694825; doi:10.1371/journal.ppat.1013733)
Supplement: Attachment — Submitted filename: Response to reviewers.pdf [file ppat.1013733.s011.pdf]

Point-by-point description, how we have addressed the Reviewers' Comments:

Reviewer's Responses to Questions

Part I - Summary

Please use this section to discuss strengths/weaknesses of study, novelty/significance, general execution and scholarship.

Reviewer #1: The research by Enz et al. is an elegant study that enhances our understanding of the impact of T3SS-2 on disrupting the epithelial barrier and modulating the inflammatory response in the gut. This is a well-planned and well-written manuscript that closely investigates the role of T3SS-2 in epithelial inflammation and the stability of the epithelial barrier. Through the application of animal models for Salmonella infection, this work provides multiple important insights and, therefore, in my opinion, is worth publishing.

Response: Thank you very much for the positive assessment of our work.

Reviewer #2: The study by Enz et al. provides valuable insights into TTSS-2-mediated gut pathology through rigorous comparison of Salmonella wild-type and TTSS-2 mutant strains across multiple mouse models. The innovative integration of immune-deficient mice model with temporal transcriptomics successfully demonstrates that TTSS-2 is essential for late-stage epithelial barrier collapse and distinct inflammatory microenvironments, which is an advance in understanding virulence-specific host manipulation. However, several conceptual and technical limitations are to be addressed to fully support the conclusions.

Response: Thank you very much for your positive assessment and the thoughtful suggestions. We have addressed them as detailed below. This has helped us to further improve our study. Thank you very much for that.

Part II – Major Issues: Key Experiments Required for Acceptance

Please use this section to detail the key new experiments or modifications of existing experiments that should be absolutely required to validate study conclusions.

Generally, there should be no more than 3 such required experiments or major modifications for a "Major Revision" recommendation. If more than 3 experiments are necessary to validate the study conclusions, then you are encouraged to recommend "Reject".

Reviewer #1:

1. For the RNASeq experiments, I did not find any information regarding RNA quality assessment (such as gels or RIN). My question is, if there is substantial disruption of the epithelial barrier in the case of STm WT, do the RNAs isolated from this group have comparable quality to those from other groups?

LINES 283-284: "Interestingly, both 24 h and 96 h S. TmSPI2-infection cluster together with 24h S. TmWT-infection, while 96 h S. TmWT-infected mucosal tissue cluster separately from all other groups (Fig 4A)."

Response:

Response: Thank you for raising this valid point. We have now added the RIN values for each sample to the supplementary data (Fig S5A) and we have added a remark to the respective results section. Lines 336-338: "All samples passed RNA quality control, with RIN values ≥8 (except for one outlier) (Fig S5A), confirming that observed differences reflect biological rather than technical variation."

Reviewer #2: Major points:

1. The manuscript identifies TTSS-2 as a driver of epithelial barrier disruption and amplified inflammation, but the underlying molecular mechanisms remain unclear. While transcriptomic data reveal differential gene expression (e.g., GsdmC downregulation), the specific TTSS-2 effectors or host signaling pathways (e.g., TNF-mediated responses) linking TTSS-2 activity to epithelial damage are not defined.

Response: We thank the reviewer for this insightful comment. We agree that further clarification of how TTSS-2 enables epithelial disruption strengthens the manuscript. We have therefore performed an additional experiment to address whether the effect of TTSS-2 virulence is in fact influenced by TTSS-2 related effector proteins. We compared 96 h infections of *S*. Tm^{SPI2} (SL13144 *ΔssaV*, that lacks a functional TTSS-2 needle and is used throughout the study) to *S*. Tm^{eff} (that retains an intact secretion system but lacks almost all SPI2 effector proteins). We added our results to the supplementary figures and explained our findings in the results section (lines 292-302, see below).

Lines 292-302: "To determine whether the effect of TTSS-2 virulence on the epithelium is mediated by the needle complex itself or by translocated effector proteins, we compared 96 h infections with *S*. Tm^{SPI2}, which lacks a functional TTSS-2 needle, and *S*. Tm^{eff}, which retains an intact needle complex but lacks all known SPI-2 effector proteins. (33,34). Ki67 staining revealed no significant differences in epithelial proliferation between the two strains after 96 h, indicating that the observed TTSS-2–dependent effects are driven by translocated effector proteins (Fig. S4 A-C)."

2. The study uses NLRC4- and CYBB-deficient mice to assess immune deficiency impacts, but this experimental design may need to be better explained. It is unclear whether other immune defects would yield similar TTSS-2 dependency, leaving the broader relevance of the observed phenotypes uncertain.

Response: We thank the reviewer for pointing out the unclarity about the broader relevance of immune deficiencies for the TTSS-2-dependent epithelial barrier disruption phenotype. To clarify, we adjust the title of this results section to more clearly define our main message. Lines 180-181: “Epithelial barrier disruption requires TTSS-2 and is not restricted to NLRC4-deficient mice.” Additionally, we updated our rationale of the experimental design. We more clearly explain that we are aiming to assess, whether higher bacterial burden within the gut tissue is linked to epithelial barrier disruption. And we did this by testing one other immune deficiency (that is not NLRC4-deficiency) that leads to increased bacterial burden as compared to immune proficient mice during *S*. Tm infection. We chose CYBB-deficient mice, because they met these criteria. CYBB-deficient mice have a defective reactive oxygen species production, which results in elevated *S*. Tm loads, and therefore serve as an independent model for uncontrolled bacterial burden. Lines 183-185: “This finding led us to question whether this phenomenon is specific to NLRC4-deficiency or if it also occurs in the context of other immune deficiencies with impaired control of bacterial burden during *S*. Tm infection.” Taken together, these results from two different models indicate that TTSS-2 driven epithelial barrier damage is not unique to NLRC4-deficency but is linked to increased bacterial burdens.

3. While testing GSDMC-KO with S. TmSPI2 was a logical first step based on the RNA-seq data, the more critical experiment would be to infect GSDMC-KO mice with S. TmWT to see if the absence of GSDMC attenuates the severe barrier disruption phenotype observed at 96hh. Besides, could GSDMC downregulation work synergistically with other pathways altered in WT iasinfection (e.g., TNF signaling)? The manuscript mentions this possibility but doesn't explore it experimentally.

Response: Thank you for raising these important points. We apologize for the lack of clarity. Our data actually show that *GsdmC* is expressed highly at 24 h p.i. after both *S*. Tm^{WT} and *S*. Tm^{SPI2} infection. Its expression remains high at 96 h p.i. after *S*. Tm^{SPI2} infection and its expression drops only after *S*. Tm^{WT} infection (Fig. 5A, S8A). Hence, GSDMC is already absent during late stages of *S*. Tm^{WT} infection, which is why we decided not to carry out the *S*. Tm^{WT} infection of GsdmC-KO mice.

We thank the reviewer for this thoughtful comment. We acknowledge that potential synergistic effects of GSDMC with other pathways were not explored in this study. However, we consider this beyond the scope of the current work, which focuses on characterizing the distinct inflammatory responses elicited by *S*. Tm^{WT} and *S*. Tm^{SPI2}. While the role of GSDMC during *S*. Tm infection remains underexplored, this question is being addressed in an ongoing, separate project in our group and will be the subject of future investigation.

4. Although the "threshold density" concept (Lines 143–144, Fig S2I) links bacterial load to epitheliale destruction, the manuscript does not explore how TTSS-2 enables surpassing this threshold. Intracellular replication assays may mechanistically anchor this observation.

Response: We appreciate the reviewer’s insightful comment and agree that our study does not mechanistically address how TTSS-2 enables bacteria to surpass the proposed threshold of tissue density for epithelial destruction. To address this point, we would like to refer to some critical previous work on the interdependence of intracellular replication and SPI2 virulence:

Extensive previous work has established that the SPI2-encoded T3SS is essential for *S*. Tm intracellular replication. Early studies identified SPI2 as a virulence locus required for systemic infection (Hensel et al., 1995, Science) and demonstrated that SPI2 encodes a second T3SS indispensable for survival and replication inside macrophages (Shea et al., 1996, PNAS). Subsequent functional genetics confirmed that SPI2 is critical for bacterial persistence in the intestine and for colitis (Hapfelmeier et al., 2005, J Immunol), and that most SPI2 effectors contribute redundantly to this process (Buckner et al., 2011, Virulence). More recent systematic studies have pinpointed the minimal effector set (sifA, sseFG, sopD2, steA, spvBCD) sufficient to restore intracellular replication and tissue colonization (Chen et al., 2021, Cell Host & Microbe). Complementary multimutant analyses revealed that SopD2 and GtgE are essential for replication and inflammation presumably by disabling the Rab32 antimicrobial pathway (Newson et al., 2024, bioRxiv). Finally, transcriptional profiling has shown that SPI2 activation is precisely timed to coincide with the onset of replication inside macrophages (Nguyen et al., 2025, Nat Microbiol). Together, these studies demonstrate that TTSS-2-mediated intracellular replication is the mechanism by which *Salmonella* expands its intracellular population, allowing it to surpass the critical threshold density that we correlate to epithelial destruction.

In summary, it is well established in the literature that SPI2 virulence enables intracellular replication of *S*. Tm within host cells. Given this consensus, we consider additional intracellular replication assays to be beyond the scope of the present study, which focuses on demonstrating that *S*. Tm^{WT} elicits a qualitatively distinct inflammatory response compared to *S*. Tm^{SPI2}. We have clarified this point throughout the results and in the discussion (lines 153-154, 225-226, 498, 500-502).

5. The timing of TTSS-2-mediated effects (72 h in immune-deficient vs. 96 h in wild-type mice) is noted, but the basis for this delay is not explained. Besides, the temporal analysis of barrier disruption provides valuable new evidence of delayed pathology in wild-type mice, but the 96hpi focus misses earlier TTSS-2 effector activity windows. Kinetic assessments (<72h) would strengthen the claim that structural collapse follows progressive barrier dysfunction.

Response: We thank the reviewer for this insightful comment. To further investigate the temporal development of epithelial barrier disruption, we performed an additional experiment analyzing wild-type C57BL/6J mice at 72 h post infection (p.i.). Consistent with the reviewer's expectation, we observed an intermediate phenotype at this time point: intestinal epithelial cell (IEC) proliferation was already reduced in *S*. Tm^{WT}-infected mice compared to the 96 h timepoint (Fig S3B). These findings indicate that epithelial pathology progresses more slowly in immunocompetent mice and that early proliferative impairment precedes structural collapse. We have added these new data and corresponding discussion to the revised manuscript (lines 282-284, 510-514).

Part III – Minor Issues: Editorial and Data Presentation Modifications

Please use this section for editorial suggestions as well as relatively minor modifications of existing data that would enhance clarity.

Reviewer #1:

1. The authors compare (S Fig. 1, S Fig. 2, etc.) different mouse models but not directly between the STm WT and STmSPI2. Throughout the text, they compare the mutant strain to the WT. In my opinion, it is worth rearranging the supplementary figures to compare two variables simultaneously—mouse strain and Salmonella strain—and applying multiple comparison statistical tests. This would allow readers to easily see the full differences in pathogen behavior and improve clarity. There is data on CFU/cecum tissue, but since there is no data on cecum weight, which may vary significantly between animals, it would be beneficial to report CFU per gram of tissue.

Given the significantly different organ loads (MLN, spleen) when comparing mouse strains, it would also be useful to compare Salmonella variants. Since the work focuses on the role of T3SS-2 in intestinal barrier stability and inflammation, clearly showing differences between STm and STmSPI2 would be advantageous.

Response: We thank the reviewer for this thoughtful suggestion. We agree that displaying the data in a format that enables direct visual and statistical comparison between *S*. Tm strains improves clarity. Accordingly, we have rearranged the supplementary figures to show *S*. Tm^{WT} and *S*. Tm^{SPI2} infection data side by side for each mouse model (see Figs S1 and S2). This allows direct comparison of infection outcomes.

Regarding the reviewer's point on normalization, we note that in this study cecum bacterial loads were expressed as CFU per total cecum tissue, following standard practice in the field (Barthel et al., 2003). Cecum weights in our experiments showed minimal variation across groups, and normalization by total cecum weight did not alter the observed trends or statistical outcomes (data not shown in manuscript, attached below). For clarity, we now mention this in the methods section (lines 623-624).

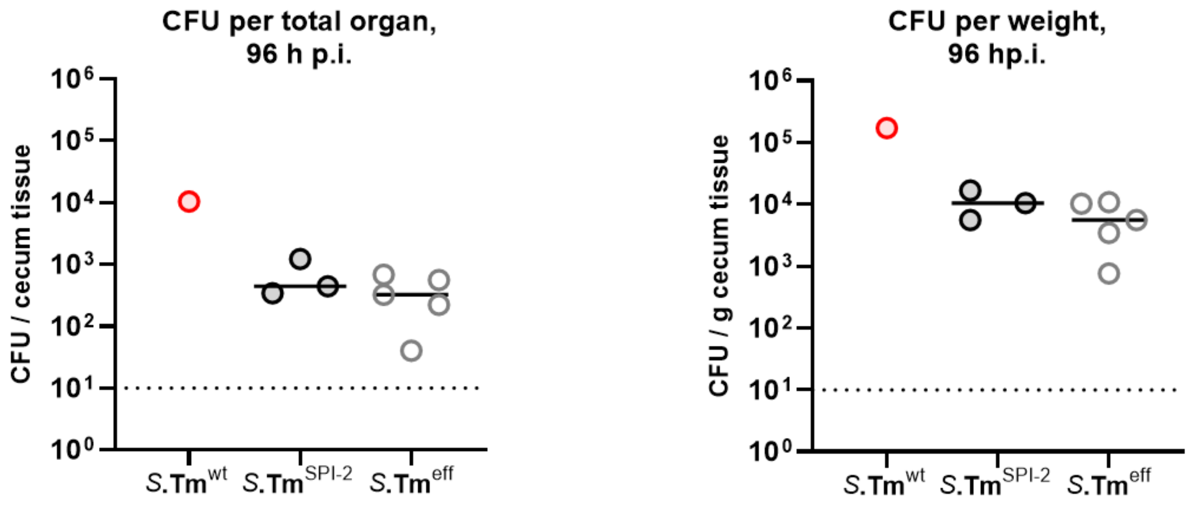

Figure 1 CFU counts of cecum tissue per total organ and per weight of wild-type mice infected with S. Tm^{WT}, S. Tm^{SPI2} or S. Tm^{eff} for 96 h to demonstrate comparable trends between the groups in both calculation methods.

Reviewer #2: Minor points:

1. While the chemotaxis exploitation hypothesis presents an intriguing dimension of TTSS-2 functionality, direct evidence linking reduced neutrophil recruitment in SPI-2 infections (Fig 4E) to impaired luminal colonization is absent.

Response: We thank the reviewer for this comment and appreciate the opportunity to clarify. We did not intend to imply direct causal link between reduced neutrophil recruitment and *S*. Tm gut luminal colonization nor to suggest that chemotaxis exploitation represents a specific TTSS-2 functionality.

The neutrophil-dependent chemotaxis phenotype is observed in the *S*. Tm^{SPI2} background (Gül et al., 2024) but is absent in *S*. Tm^{WT} infections, highlighting qualitative differences in the inflammatory environment elicited by these strains. The Tsr^{-} vs. Tsr^{+} competition experiment mentioned in the discussion serves as a conceptual example of how these differences can influence experimental outcomes, rather than reflecting a direct TTSS-2-dependent mechanism. To prevent potential misunderstanding, we have clarified this in the discussion (lines 576-578).

2. The assessment of gut inflammation relies heavily on Lcn-2 levels and Ki67 staining, but additional markers would strengthen phenotypic characterization.

Response: We thank the reviewer for raising this point. We agree that including additional inflammation markers strengthens the manuscript. In our main characterization, comparing *S*. Tm^{WT} and *S*. Tm^{SPI2} induced inflammation in wild-type mice at 96 h p.i., we already assessed eight different inflammation markers: fecal lipocalin-2 levels, quantification of expelled IECs, crypt length, IEC proliferation, TNF concentration, and neutrophil and macrophage infiltration, complemented by RNAseq analysis revealing pro-inflammatory gene expression patterns.

To further validate these observations, we have now performed qPCR validation of selected pro-inflammatory genes identified in the RNAseq dataset (Fig S5D; lines 349-351). Additionally, we performed HE staining to demonstrate cecum tissue histopathology scores in NLRC4-proficient and -deficient mice (Fig S1 G, H; line 141, 156-157). These data further confirmed the inflammatory phenotypes described above.

3. Besides cecum, other intestinal regions (e.g., colon, ileum) are not assessed, leaving uncertainty about whether TTSS-2 effects are cecum-specific.

Response: We agree with the reviewer that we cannot exclude the possibility of TTSS-2 effects on different intestinal regions than the cecum. However, in the streptomycin mouse model of *Salmonella* infection that was established in the field 2003 by Barthel et al., traditionally the cecum is used to analyse histopathology as it represents the primary site of *S*. Tm invasion. To ensure comparability between previous studies, we likewise focused our analyses on the cecum in this work. To clarify this, we updated the explanation in the material and methods section (Lines 614-615).

4. The sample sizes for CYBB experiments (Fig 2C, E; n=3-4) may lead to unreliable conclusions, necessitating either statistical justification for the current sample size or an increase in the number of specimens.

Response: Thank you for raising this point. As noted in the manuscript (lines 214-215 and 494-495), we did not increase the sample size because CYBB-deficient mice experienced considerable disease burden following *S*. Tm^{WT} infection. Consequently, we present these findings as “trends” rather than a statistically supported difference (lines 214-215). We hope the reviewer will appreciate our ethical decision to avoid additional animal suffering while still transparently reporting the observed pattern.

5. Cytokine analysis is limited to TNFα (Fig S3C); measuring additional cytokines such as IL-1β, IFNγ in cecal lysates would provide a more comprehensive picture of the inflammatory response.

Response: We appreciate the reviewer’s suggestion to include additional cytokine measurements such as IL-1β and IFNγ to provide a more comprehensive view of the inflammatory response. While we did not perform multiplex cytokine quantification, we agree that transcriptional profiling can provide complementary information on inflammatory signalling. To this end, we have expanded our analysis by including qPCR data for key cytokine and immune-related genes (Fig S5D; lines 349-351). These data support our TNFα results and further indicate that the inflammatory profile remains attenuated in the TTSS-2 mutant infection.

6. RNA-seq identified DEGs (such as GsdmC in Fig 4C) would require more validation via qPCR or WB due to their mechanistic significance.

Response: Thank you for this suggestion, we agree that confirmation of certain DEGs with a second method would strengthen the manuscript. As mentioned above, we have performed qPCR validation of selected pro-inflammatory genes identified in the RNAseq dataset (Fig S5D; lines 316-318). Additionally, we performed qPCR analysis of the GsdmC2 and GsdmC4 genes and found the same expression pattern as identified by RNAseq (Fig S8A; line 416).

7. Unclear rationale for including S. TmAvir strains in transcriptomics (Lines 275-277) without results discussion.

Response: Thank you for pointing out this unclarity in the text. We extended the explanation of our rationale for including the *S*. Tm^{AVIR} strain (Lines 331-334).

8. When quantifying "dislodged enterocytes" (Fig 3D), the criteria for defining an "expelled IEC" (e.g., morphological features or marker co-staining) are not specified, making the quantification method ambiguous

Response: Thank you for pointing out this unclarity. We extended the material and methods section with a microscopy image to demonstrate our quantification methods (Fig S9; lines 646-648).

PLOS authors have the option to publish the peer review history of their article (what does this mean?). If published, this will include your full peer review and any attached files.

If you choose "no", your identity will remain anonymous but your review may still be made public.

Do you want your identity to be public for this peer review? For information about this choice, including consent withdrawal, please see our Privacy Policy.

Reviewer #1: No

Reviewer #2: No

[NOTE: If reviewer comments were submitted as an attachment file, they will be attached to this email and accessible via the submission site. Please log into your account, locate the manuscript record, and check for the action link "View Attachments". If this link does not appear, there are no attachment files.]

Figure resubmission:

While revising your submission, please upload your figure files to the Preflight Analysis and Conversion Engine (PACE) digital diagnostic tool, https://pacev2.apexcovantage.com/. PACE helps ensure that figures meet PLOS requirements. To use PACE, you must first register as a user. Registration is free. Then, login and navigate to the UPLOAD tab, where you will find detailed instructions on how to use the tool. If you encounter any issues or have any questions when using PACE, please email PLOS at figures@plos.org. Please note that Supporting Information files do not need this step. If there are other versions of figure files still present in your submission file inventory at resubmission, please replace them with the PACE-processed versions.

Reproducibility:

To enhance the reproducibility of your results, we recommend that authors of applicable studies deposit laboratory protocols in protocols.io, where a protocol can be assigned its own identifier (DOI) such that it can be cited independently in the future. Additionally, PLOS ONE offers an option to publish peer-reviewed clinical study protocols. Read more information on

sharing protocols at https://plos.org/protocols?utm_medium=editorial-email&utm_source=authorletters&utm_campaign=protocols
